# The Prognostic Landscape of Tumor-Infiltrating Immune Cells and Immune Checkpoints in Glioblastoma

**DOI:** 10.1177/1533033819869949

**Published:** 2019-08-26

**Authors:** Shiman Wu, Wenli Yang, Hua Zhang, Yan Ren, Ziwei Fang, Chengjie Yuan, Zhenwei Yao

**Affiliations:** 1 Department of Radiology, Huashan Hospital, Fudan University, Shanghai, P.R. China; 2 Pathology department, Jiangmen Central Hospital, Affiliated Jiangmen Hospital of Sun Yat-sen University, Jiangmen, P.R. China; 3 Department of Radiology, the Affiliated Hospital of Qingdao University, Qingdao, P.R. China; 4 Department of Orthopedics, Huashan Hospital, Fudan University, Shanghai, P.R. China

**Keywords:** TCGA, TCIA, glioblastoma, GBM, immunotherapy, checkpoint therapy

## Abstract

Tumor-infiltrating immune cells are part of a complex microenvironment and associated with improved clinical outcomes in a broad range of tumor types. However, a detailed map for the prognostic landscape of tumor-infiltrating immune cells and immune checkpoint modulators in glioblastoma is still lacking. Here, with the web-accessible resource, The Cancer Immunome Archive, 28 types of both adaptive and innate tumor-infiltrating immune cells were characterized in glioblastoma. Tumors lacking central memory CD4 T cells or natural killer cells were associated with better prognosis in glioblastoma, as verified by immunohistochemical analysis. Moreover, Kaplan-Meier analysis for a total of 71 key immune checkpoint molecules revealed that the expression level of inducible T cell costimulators, tumor necrosis factor superfamily member 14, and UL16 binding protein 1 were negatively correlated with the clinical outcome of patients with glioblastoma. In addition, there was a significant difference between nontumor and glioblastoma samples of several immune checkpoint modulators based on the expression level of their corresponding gene. Collectively, the annotation of tumor-infiltrating immune cells and immune checkpoint modulators in glioblastoma provides a valuable resource for identifying their involvement in tumor escape mechanisms and response to therapy.

## Introduction

Glioblastoma, also known as glioblastoma multiforme (GBM), is the most prevalent and malignant primary brain tumor associated with an extremely aggressive clinical course and poor prognosis.^1^ For newly diagnosed patients, the standard therapy involves aggressive resection and radiation as well as temozolomide), but the median overall survival (OS) remains a dismal 15 to 17 months.^2^ Therefore, there is an urgent need to develop novel and effective therapeutic approaches for GBM.^3^ Fortunately, recent years have witnessed exciting breakthroughs in novel immune strategies, which boost the body’s anticancer immune responses instead of directly targeting tumor cells.^4^ Accumulating studies of immunotherapy for various tumors have brought new knowledge and new hope for improving the prognosis of GBM.^5^ Immunohistochemistry is the most common technique used to analyze the immune cell composition of tumors but is limited as only a few immune cells can be evaluated at once.^6^ With the increasing genomic data and rapid development of bioinformatics, it is now possible to computationally mine the data for immunological insights.

Tumor-infiltrating immune cells, as part of a complex microenvironment, can suppress the tumor or provide support for tumor growth based on the type of cells and their functional interactions.^7^ Typical immune cells in the tumor include T lymphocytes, natural killer (NK) cells, macrophages, dendritic cells (DC), polymorphonuclear leukocytes, and occasional B cells.^8^ In recent years, the importance of the immune infiltrate as a prognostic marker has become increasingly relevant.^9^ The high expression level of intraepithelial T lymphocytes in ovarian was demonstrated to associate with better OS compared with ovarian cancer without lymphocytes.^10^ Tumor-infiltrating NK cells and Th1 markers were associated with increased OS, for example, HLA-DRC and CXCR3C T cells; whereas a high number of T cells, especially with high CD69 expression correlated with a poorer prognosis in renal cancer.^11^

Immune checkpoints are activity modulation of T cells by costimulatory and coinhibitory molecules to achieve an optimal immune response.^3^ Immunostimulators are downregulated to avoid immune destruction, while immunoinhibitory genes are upregulated to facilitate tumor escape.^12^ Over the past years, immune checkpoints with antibodies that target programmed cell death protein 1 (PD-1), cytotoxic T lymphocyte-associated antigen 4 (CTLA-4), and indoleamine 2,3-dioxygenase (IDO) have showed remarkable success in several tumors such as melanoma,^13^renal cancer,^14^ and non-small cell lung cancer.^15^ For example, 2 checkpoint inhibitors that target PD-1, pembrolizumab and nivolumab, were approved for metastatic melanoma by the Food and Drug Administration.^16^CTLA-4 , also known as CD152, was identified to competitively bind to B7 and block costimulatory signals.^17^ In addition, CTLA-4 can lead to dysregulation of FoxP3^+^ regulatory T cells (Tregs) and is related to B cells reduction in lymphoid organs through the increase in autoreactive CD21 B cells.^18,19^

**Figure 1.**
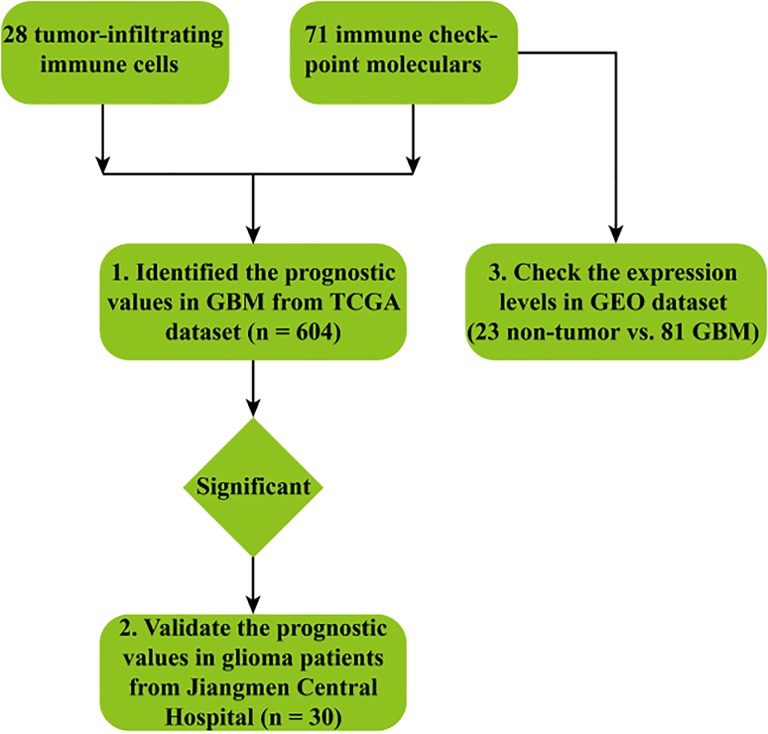
The workflow of our study.

However, a comprehensive view of the immune cell landscape in GBM is still lacking. Here, using the web-accessible resource, The Cancer Imaging Archive (TCIA), 28 types of both adaptive and innate tumor-infiltrating immune cells were characterized in GBM based on The Cancer Genome Atlas (TCGA) data sets. In addition, a total of 71 immune checkpoint molecules were also determined and their expression profile was further analyzed in nontumor and GBM samples to validate the bioinformatics results. The overall workflow of our study was shown in Figure 1. Collectively, the data demonstrated that the metagene approach by TCIA provides valuable information about the tumor–immune cell interactions.

## Materials and Methods

### Cancer Expression Profiling Data

The Cancer Genome Atlas data of GBM (n = 604) were downloaded from Firebrowse (http://firebrowse.org/) and used to evaluate the expression of tumor-infiltrating immune cells and checkpoints in patients with GBM by the following bioinformatics analysis. The expression data quantified as RSEM (RNA-Seq by expectation-maximization) was logarithmically transformed. The gene expression profile of GSE4290 downloaded from the Gene Expression Omnibus (GEO) database (http://www.ncbi.nlm.nih.gov/geo/) was used to evaluate the expression profile of checkpoints in nontumor and GBM samples.^20^ In this data set, 23 brain tissue samples from patients with epilepsy were used as nontumor samples, 81 GBM samples were selected for more detailed characterization. The messenger RNA expression data was based on the GPL570 platform (Affymetrix Human Genome U133 Plus 2.0 Array).

### Evaluation of Tumor-Infiltrating Immune Cells and Checkpoints

The enrichment of immune cell types overrepresented in the tumor microenvironment (TME), within patients, was evaluated by single sample gene set enrichment analysis (ssGSEA).^21^ Gene set enrichment analysis (GSEA)is a powerful analytical method used to interpret gene expression data.^22^ The method derives its power by focusing on gene sets, groups of genes that share common biological function, chromosomal location, or regulation.^22^ Enriched immune cell types with a normalized enrichment score >0 are illustrated as bubble plots, in which the size represents the percentage of patients with the enriched cell type. In this study, the percentage and prognostic value (hazard ratio) of tumor-infiltrating immune cells and checkpoints were evaluated in TCGA data set by TCIA (https://tcia.at/). The web-accessible resource TCIA, which is mainly based on the metagenes approach, was developed to allow researchers to dissect tumor–immune cell interactions and identify prognostic or predictive markers.^23^ The method was successfully validated by Fluorescence Activated Cell Sorting(FACS) and used for the determination of the immune cell landscapes in colorectal cancer,^24^ liver,^25^ and lung cancer.^26^

### Patients and Specimens

Tumor samples were collected from patients who were operated at Jiangmen Central Hospital, Affiliated Jiangmen Hospital of Sun Yat-sen University (Jiangmen, China). A total of 30 samples of different grades of astrocytomas were used in this study. The clinical characteristics of patients with glioma are listed in Table 1. The study was approved by the Ethics Committees of Jiangmen Central Hospital, Affiliated Jiangmen Hospital of Sun Yat-sen University, and all patients gave written informed consent.

**Table 1.** Clinical Characteristics of Patients With Glioma.

Age (years)	
Median	46
Range	6-74
Sex	
Male	15
Female	15
Tumor size (cm)	
Median	4.7
Range	1-7.9
Tumor localization	
Left hemisphere	16
Right hemisphere	15
WHO grade	
I	3
II	15
III	6
IV	6
Histologic subtype	
Gliocytoma	11
Glioblastoma	6
Astrocytoma	5
Others	8
Total	30

### Immunohistochemical Staining

Immunohistochemical staining was performed using a standard immunoperoxidase staining procedure. Antibodies against CD56, CD4, inducible T cell costimulators (ICOS), tumor necrosis factor superfamily member 14 (TNFSF14), and UL16 binding protein 1 (ULBP1) were used at 1:100 dilutions. The expression of ICOS, TNFSF14, and ULBP1 in malignant specimens was evaluated according to the methods described by Pinheiro. ICOS, TNFSF14, and ULBP1 expression were semiquantitatively scored for the extent of immunoreactivity as follows: 0 is <5% immunoreactive cells; 1 is 5% to 25% immunoreactive cells; 2 is 25% to 50% immunoreactive cells; 3 is 50% to 75% immunoreactive cells; and 4 is >75% immunoreactive cells. Additionally, the staining intensity was semiquantitatively scored as 0 (negative), 1 (weak), 2 (intermediate), or 3 (strong). The final immunoreaction score was defined as the product of both parameters (extension and intensity). The expression of CD56 and CD4 in malignant specimens was evaluated according to the positive cell count per high-power field. Cells stained positive for the indicated markers were counted on the 20× (objective) images of tumor nodules of similar size. Comparisons of wild-type and mutation groups were performed using *t* tests.

### Statistical Analysis

The prognostic value of the tumor-infiltrating immune cells and immune checkpoint molecules was estimated by Kaplan-Meier analysis and evaluated by the log-rank test. A *P* value <.05 was considered significant. The differences in immune checkpoint molecules between nontumor and GBM samples were assessed using the Wilcoxon test (***P < .001, **P < .01, *P < .05). Statistical analysis was performed in R language (version: 3.3.3; https://www.r-project.org/).

## Results

### The Prognostic Value of Tumor-Infiltrating Immune Cells

**Figure 2.**
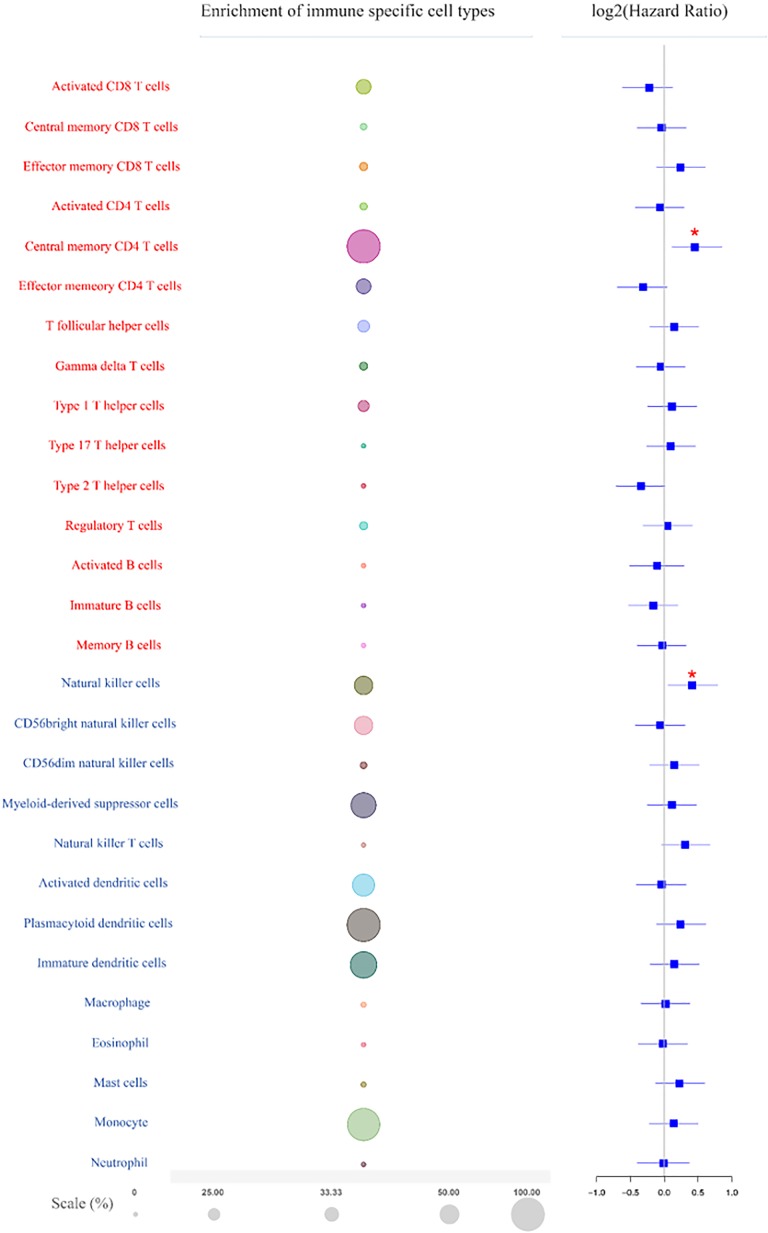
The enrichment and prognostic value of tumor-infiltrating immune cells in GBM. Left panel, Twenty-eight types of adaptive and innate immune cells. Red represents adaptive immune cells; blue represents innate immune cells. Middle panel, Bubble plot shows enrichment of the adaptive and innate immune cells. The size of the circles indicates the percentage of patients, false discovery rate (FDR) < 0.1. Right panel, Kaplan-Meier analysis of the prognostic value of the adaptive and innate immune cells in GBM. Statistical significance was determined by the Wilcoxon test (*** *P* < .001, **P < .01, *P < .05). GBM indicates glioblastoma.

High resolution of the landscape of the immune cell is required to dissect tumor–immune cell interactions and identify prognostic and predictive markers. Twenty-eight types of tumor-infiltrating immune cells including 15 major types related to adaptive immunity and 13 types related to innate immunity were estimated based on TCIA database (Figure 2; left panel). The adaptive immune cell types including activated CD8 T cells, central memory CD8 T cells, effector memory CD8 T cells, activated CD4 T cells, central memory CD4 T cells, effector memory CD4 T cells, T follicular helper (Tfh) cells, gamma delta T (Tgd) cells, type 1 T helper cells, type 17 T helper cells, type 2 T helper cells, Treg cells, activated B cells, immature B cells, and memory B cells. The innate immune cell types comprised NK cells, CD56bright natural killer cells, CD56dim natural killer cells, myeloid-derived suppressor cells, NK T cells, activated DCs, plasmacytoid DCs, immature DCs, macrophages, eosinophils, mast cells, monocytes, and neutrophils. Enrichment of the immune cells showed that adaptive immune central memory CD4 T cells, which were enriched in all patients, were the most abundant cell type in GBM (Figure 2; middle panel). The innate immune plasmacytoid DCs and monocytes were also abundant, being enriched in 98.7% and 96.7% patients, respectively. The rest of the immune cell types were all enriched in less than 90% patients (Figure 2; middle panel).

**Figure 3.**
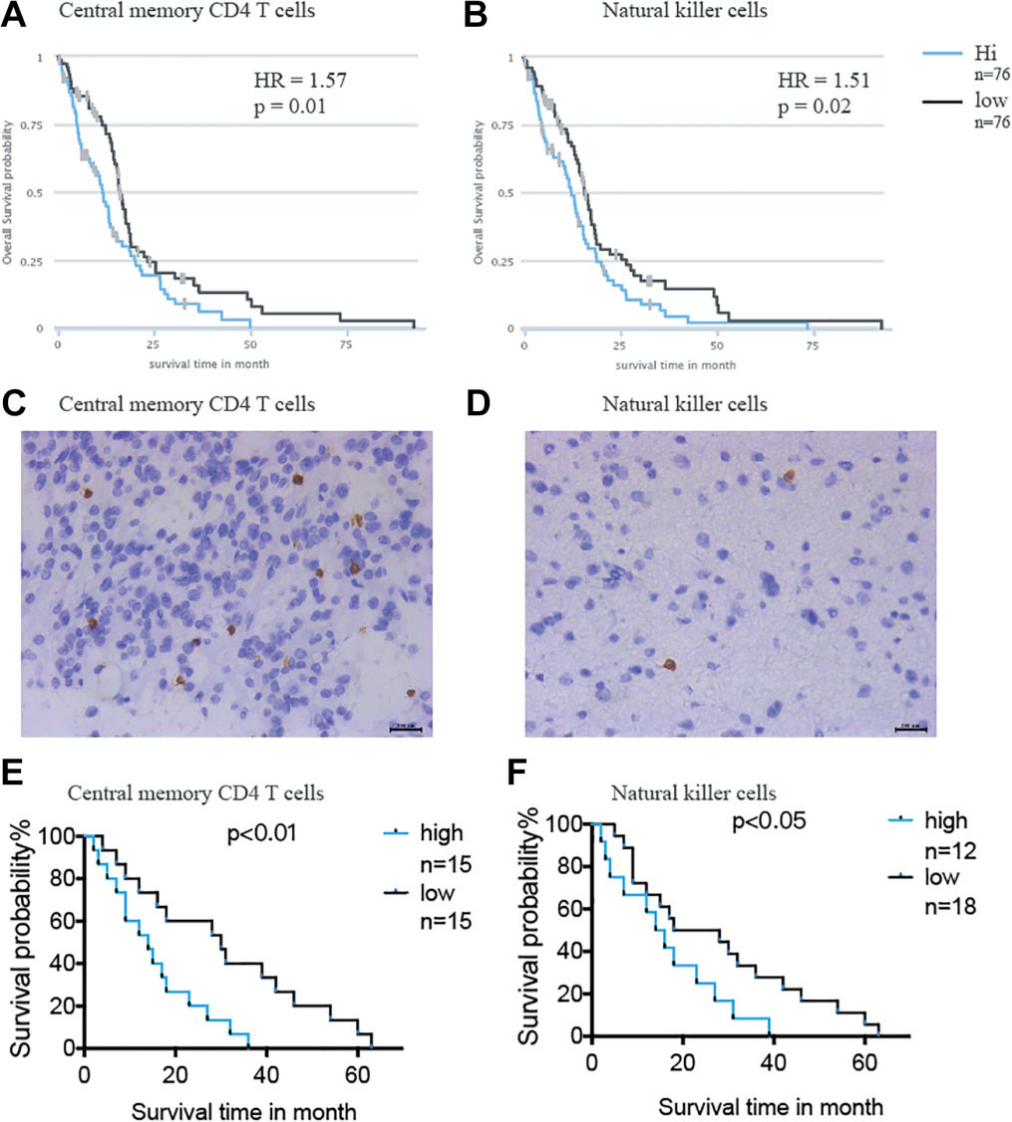
Evaluation of adaptive immune cell type central memory CD4 T cells and innate immune cell type NK cell in glioma samples. The Kaplan-Meier survival analysis of the (A) central memory CD4 T cells and (B) NK cells in TCGA data set. Immunohistochemistry staining shows the express level of (C) central memory CD4 T cell and (D) NK cell in patients with glioma collected from hospital. The Kaplan-Meier survival analysis of the (E) central memory CD4 T cells and (F) NK cell in patients with glioma collected from hospital. NK cells indicate natural killer cells; TCGA, The Cancer Genome Atlas.

The prognostic value of the immune cells was evaluated by Kaplan-Meier analysis through TCIA (Figure 2; right panel). Tumors lacking adaptive immune cell type central memory CD4 T cells (Figure 3A) and the innate immune cell type NK cells (Figure 3B) were associated with better OS probability. Immunohistochemical staining was performed to examine the expression of central memory CD4 T cells (Figure 3C) and NK cells (Figure 3D) in 30 glioma samples from patients. Log-rank analysis of the Kaplan-Meier survival curves was consistent with the results predicted by TCIA, further demonstrating the functions of central memory CD4 T cells (Figure 3E) and NK cells in patients with glioma (Figure 3F).

### The Prognostic Value of Immune Checkpoint Molecules

**Figure 4.**
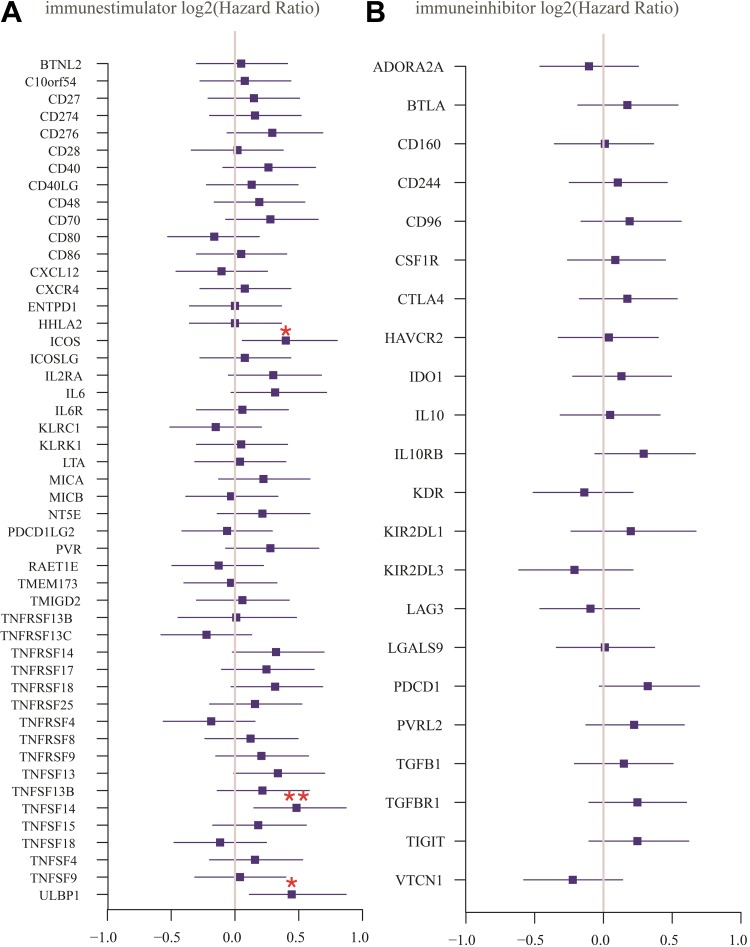
Kaplan-Meier analysis for the prognostic value of immune stimulators (A) and immune inhibitors (B) in GBM. Statistical significance was determined by the Wilcoxon test (***P < .001, **P < .01, *P < .05). GBM indicates glioblastoma.

**Figure 5.**
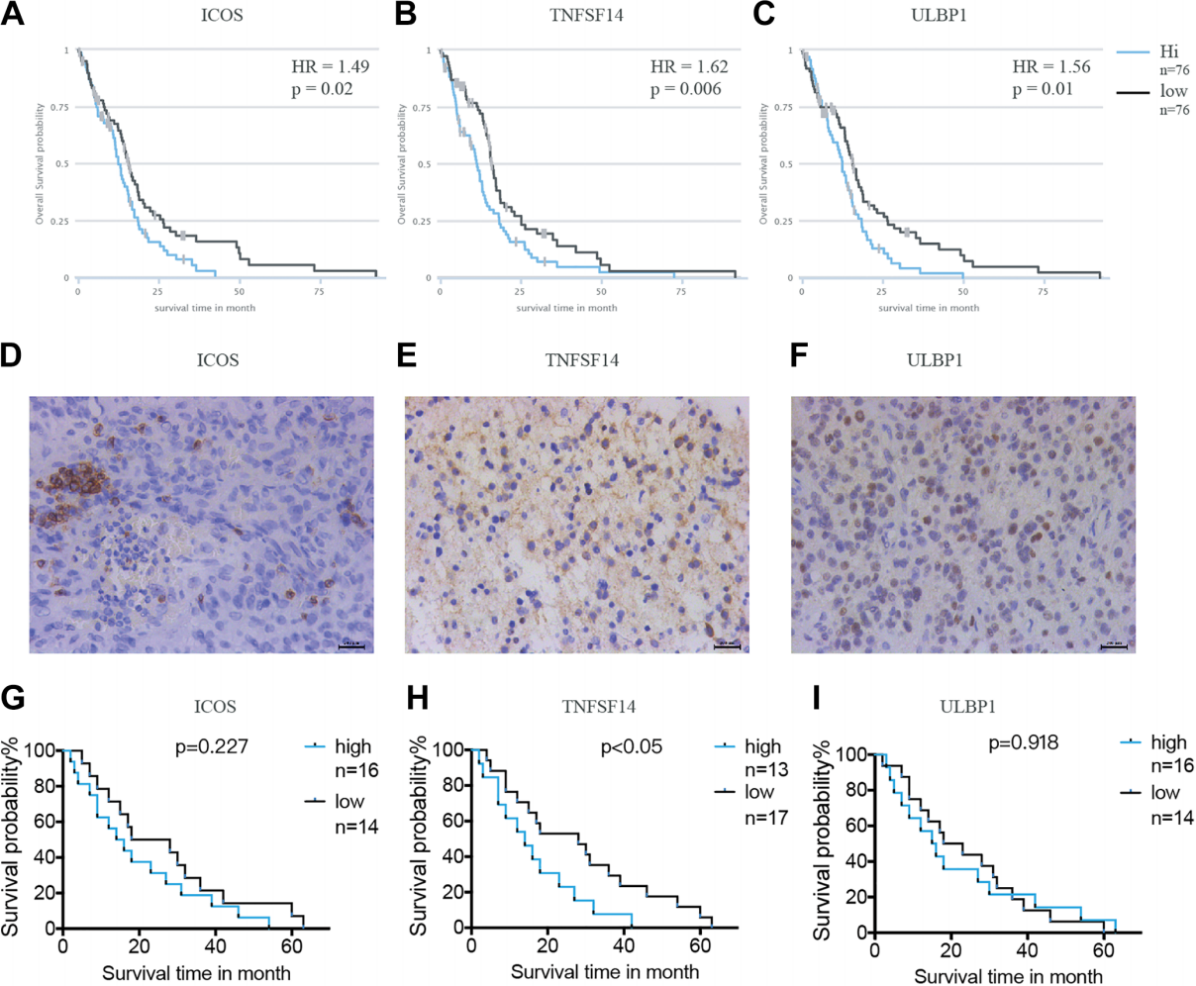
Evaluation of checkpoint molecules ICOS, TNFSF14, and ULBP1 in glioma samples. Kaplan-Meier survival analysis of the checkpoint molecules (A) ICOS, (B) TNFSF14, and (C) ULBP1 in TCGA data set. Immunohistochemical staining showing the expression level of checkpoints (D) ICOS, (E) TNFSF14, and (F) ULBP1 in patients with GBM and (G) ICOS, (H) TNFSF14, and (I) ULBP1 in patients with glioma collected from hospital. GBM indicates glioblastoma; ICOS, inducible T cell costimulators; TNFSF 14, tumor necrosis factor superfamily member 14; ULBP1, UL16 binding protein 1.

Costimulatory and coinhibitory molecules have a pivotal role in the immune system, as they determine the functional outcome of T-cell receptor signalling.^27,28^ First, the prognostic value of 71 key immune modulators was evaluated, including 49 immune stimulators (Figure 4A) and 22 immune inhibitors (Figure 4B) by Kaplan-Meier survival analysis in TCIA database. The results revealed that 3 immune stimulators including ICOS (Figure 5A), TNFSF14 (Figure 5B), and ULBP1 (Figure 5C) were negatively correlated with the clinical outcome of patients with GBM, while no significant differences were found in the clinical outcome between low and high expression of immune inhibitors. The expression of ICOS (Figure 5D), TNFSF14 (Figure 5E), and ULBP1 (Figure 5F) was assessed by immunohistochemistry in patients with glioma, confirming that TNFSF14 was associated with the clinical outcome of patients with glioma (Figure 5G). The other 2 checkpoints ICOS (Figure 5H) and ULBP1 (Figure 5I) were not significantly correlated with the survival probability, possibly due to insufficient samples.

### The Expression Profile of Immune Checkpoint Molecules

**Figure 6.**
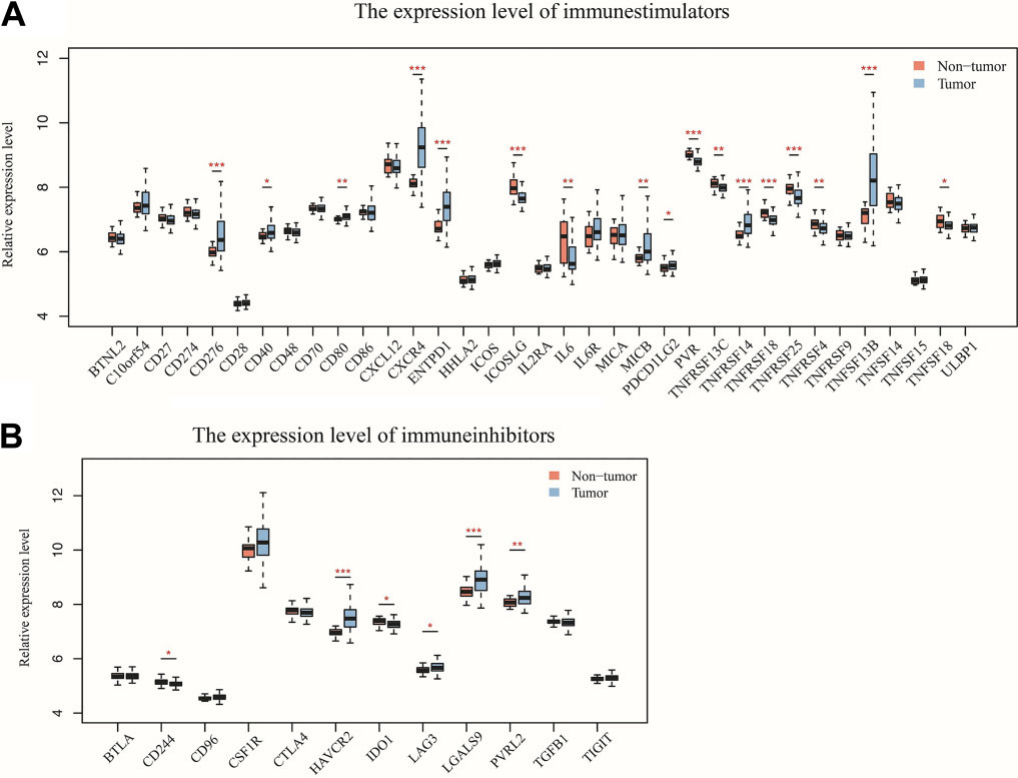
The expression level of (A) immune stimulators and (B) immune inhibitors in nontumor and GBM samples. Statistical significance was determined by the Wilcoxcon test (***P < .001, **P < .01, *P < .05). GBM indicates glioblastoma; TCGA, The Cancer Genome Atlas.

The differentially expressed immune checkpoint molecules may be potential targets for further clinical research. Further comparison of the expression of several key immune molecules in nontumor and GBM samples downloaded from GEO database was performed. Among the immune stimulators, the expression level of CD276, CD40, CD80, CXCR4, ENTPD1, MICB, PDCD1LG2, TNFRSF14, and TNFSF13B was significantly increased in GBM samples compared to nontumor samples (Figure 6A). In contrast, the expression level of ICOSLG, IL6, PVR, TNFRSF13C, TNFRSF18, TNFSF25, TNFRSF4, and TNFSF18 was significantly decreased in GBM samples (Figure 6A). Regarding the immune inhibitors, HAVCR2, LAG3, LGALS9, and PVRL2 were significantly more highly expressed in GBM samples compared with nontumor samples, while CD244 and IDO1 showed an opposite expression pattern (Figure 6B).

## Discussion

Glioblastoma is the most prevalent and malignant tumor in the central nervous system (CNS).^1^ Cancer immunotherapy aims to generate an efficacious therapeutic modality to enhance the specificity and power of the immune system to combat tumors.^29^ Indeed, immunotherapy has shown remarkable success in the treatment of multiple solid and hematological cancers.^29,4^ Although the CNS has been traditionally considered as an “immune privileged” organ, it is now regarded as a promising target for cancer immunotherapy since there is accumulating evidence that the CNS actively communicates with the immune system.^3^ Recent breakthroughs in cancer immunotherapy and rapid development of high-throughput technologies have sparked intensive research into immunology using bioinformatics tools.^30^ In the present study, the prognostic landscape of tumor-infiltrating immune cells and immune checkpoint molecules was comprehensively characterized in GBM based on the TCGA data sets using the analytic tools from TCIA (Figure 1), providing valuable information about the tumor–immune cell interactions.

Interactions between tumor and immune cells in the TME have a crucial role in tumor progression and treatment response.^7^ The results showed that adaptive immune cell type central memory CD4 T cells, enriched in all patients, were associated with poor prognosis in GBM. Central memory CD4 T cells have been characterized by their capacity of proliferation and differentiation into effector memory CD4 T cells.^31^ A previous study revealed that homeostasis of central memory CD4 T cells is a key factor to sustain the asymptomatic stage of human immunodeficiency virus type 1 (HIV-1) infection and central memory CD4 T cells homeostatic failure is responsible for progression to acquired immunodeficiency syndrome.^32^ It has also been demonstrated that central memory CD4 T cells are associated with incomplete restoration of the CD4 T cell pool after treatment-induced long-term undetectable HIV viraemia.^33^ The present study is the first to report the association of central memory CD4T cells with GBM. Unexpectedly, it was also found that the innate immune cell type NK cells, enriched in 48% of patients, negatively associated with clinical survival probability in GBM. Natural killer cells, large granular lymphocytes, are able to directly lyse infected or transformed cells without specific immunization.^7^ The activities of NK cells are regulated by the interactions of various receptors expressed on their surfaces with cell surface ligands in both viral and tumor models.^34^ Interactions between NK cells and DCs, T cells, and B cells also dramatically alter the overall immune response to cancer. Although NK cells have been demonstrated to act as direct antitumor agents or stimulate the endogenous cytotoxicity in some cancers, our findings revealed that the lack of NK cells was associated with better OS probability in patients with GBM.

Cancer immunotherapies with antibodies that target immune checkpoint molecules have demonstrated therapeutic efficacy and durable response for several tumor types.^30^ The prognostic values of 71 key immune checkpoint molecules were determined in GBM by Kaplan-Meier analysis, showing that the expression level of 3 immune stimulators—ICOS, TNFSF14, and ULBP1, which were negatively correlated with the clinical outcome of patients with GBM. ICOS form homodimers and play an important role in cell–cell signaling, immune responses, and regulation of cell proliferation.^35^ Due to its dual role in sustaining T cell activation and effector functions, as well as its association with Treg suppressive activity, targeting ICOS/ICOS-L represents an attractive approach in enhancing antitumor immunity.^36^ The TNFSF14 (best known as LIGHT), which is expressed by activated T cells, serves as a key component of the communication system that controls the response of T cells.^37^ A recent study revealed that increased expression of TNFSF14 can increase T-cell proliferation, activation, and infiltration, resulting in enhanced tumor-specific immune-mediated tumor regression in primary tumors and colorectal liver metastases.^38^ ULBP1 expressed on the tumor cell surface binds to the natural killer group 2 member D (NKG2D) receptor, an immune system-activating receptor on NK cells and T cells.^39^ The interactions between ULBP1 and NKG2D have been demonstrated to improve OS in patients with gastric cancer.^40^ Immunotherapy with checkpoint inhibitors in some tumors showed remarkable success in recent years.^41^ Several clinical trials of checkpoint inhibitors are ongoing in GBM and other brain carcinomas.^3^ Unfortunately, no immune inhibitor was found to significantly associate with the survival probability of patients with GBM in this study, possibly due to the limited number of samples. Furthermore, several immune checkpoint modulators were shown to be significantly different between non-tumor and GBM samples based on the expression level of their corresponding gene. The immune checkpoint molecules may be useful as prognostic biomarkers with the potential to improve clinical outcomes of patients with GBM.

In conclusion, this comprehensive analysis of 28 types of tumor-infiltrating immune cells and 71 immune checkpoint molecules in GBM not only revealed valuable information about tumor–immune cell interactions but also provided critical insight into new immunotherapeutic strategies and potential new predictive biomarkers.

## Abbreviations

CNS: central nervous system
CTLA-4: cytotoxic T lymphocyte-associated antigen 4
DC: dendritic cells
GBM: glioblastoma
GEO: Gene Expression Omnibus
ICOS: inducible T cell costimulators
IDO: indoleamine 2,3-dioxygenase
NK: natural killer
OS: overall survival
PD-1: programmed cell death protein 1
TCIA: The Cancer Imaging Archive
TCGA: The Cancer Genome Atlas
TME: tumor microenvironment
TNFSF14: tumor necrosis factor superfamily member 14
Treg: regulatory T cells
ULBP1: UL16 binding protein 1.
